# Non-Destructive Techniques Based on Eddy Current Testing

**DOI:** 10.3390/s110302525

**Published:** 2011-02-28

**Authors:** Javier García-Martín, Jaime Gómez-Gil, Ernesto Vázquez-Sánchez

**Affiliations:** 1 Department of Signal Theory, Communications and Telematic Engineering, University of Valladolid (UVA), 47011 Valladolid, Spain; E-Mails: jgomez@tel.uva.es (J.G.-G.); ernesto.vazquez@uva.es (E.V.-S.); 2 Ingeniería y Sistemas de Ensayos no Destructivos (ISEND), Ed. Galileo Azul, 103, 47151 P. T. Boecillo Valladolid, Spain

**Keywords:** non-destructive testing, eddy current, magnetic field, sensor, coil probe, impedance, crack, impedance plane, conductivity

## Abstract

Non-destructive techniques are used widely in the metal industry in order to control the quality of materials. Eddy current testing is one of the most extensively used non-destructive techniques for inspecting electrically conductive materials at very high speeds that does not require any contact between the test piece and the sensor. This paper includes an overview of the fundamentals and main variables of eddy current testing. It also describes the state-of-the-art sensors and modern techniques such as multi-frequency and pulsed systems. Recent advances in complex models towards solving crack-sensor interaction, developments in instrumentation due to advances in electronic devices, and the evolution of data processing suggest that eddy current testing systems will be increasingly used in the future.

## 1. Introduction

Non-destructive techniques are used in the metal industry and science in order to evaluate the properties of a wide variety of materials without causing damage. Some of the most common non-destructive techniques are electromagnetic, ultrasonic and liquid penetrant testing. One of the conventional electromagnetic methods utilized for the inspection of conductive materials such as copper, aluminum or steel is eddy current non-destructive testing [1].

Electromagnetic methods such as eddy current, magnetic particle or radiographic and ultrasonic methods all introduce electromagnetic or sound waves into the inspected material in order to extract its properties. Penetrant liquid techniques can detect cracks in the test material by using either fluorescent or non-fluorescent dyes. In addition to these methods, scientists such as Shujuan *et al.* [2], Noorian *et al.* [3] and Aliouane *et al.* [4] have researched non-destructive testing based on a combination of electromagnetic and sound waves using electromagnetic acoustic transducers, best known as EMATs.

The principle of the eddy current technique is based on the interaction between a magnetic field source and the test material. This interaction induces eddy currents in the test piece [1]. Scientists can detect the presence of very small cracks by monitoring changes in the eddy current flow [5].

This paper reviews non-destructive eddy current techniques that permit high-speed testing [6] of up to 150 m/s [7] under harsh operating conditions where other techniques cannot be used. Eddy current testing is especially fast at automatically inspecting semi-finished products such as wires, bars, tubes or profiles in production lines. The results of eddy current testing are practically instantaneous, whereas other techniques such as liquid penetrant testing or optical inspection require time-consuming procedures that make it impossible [8], even if desired, to inspect all production.

Eddy current testing permits crack detection in a large variety of conductive materials, either ferromagnetic or non-ferromagnetic, whereas other non-destructive techniques such as the magnetic particle method are limited to ferromagnetic metals. Another advantage of the eddy current method over other techniques is that inspection can be implemented without any direct physical contact between the sensor and the inspected piece.

In addition, a wide variety of inspections and measurements may be performed with the eddy current methods that are beyond the scope of other techniques. Measurements of non-conductive coating thickness [9] and conductivity can be done. Conductivity is related to the composition and heat treatment of the test material. Therefore, the eddy current method can also be used to distinguish between pure materials and alloy compositions and to determine the hardness of test pieces after heat treatments [8].

Since the 1950s the role of eddy current testing has developed increasingly in the testing of materials, especially in the aircraft [10] and nuclear industries [11]. The extensive research and development in highly sensitive eddy current sensors and instruments over the last sixty years indicates that eddy current testing is currently a widely used inspection technique.

This paper presents the basis of non-destructive eddy current testing and provides an overview of the research conducted by many authors who continue to develop this technique. The fundamentals of eddy current inspection and the main variables of this technique are presented in Sections 2 and 3. Section 4 reviews the state-of-the-art sensors and research. Section 5 reviews the state of modern equipment, and Section 6 presents the applications and research trends of eddy current inspection. Finally, Section 7 presents a discussion of eddy current testing.

## 2. Principles of Operation of Eddy Current Testing

The objective of this section is to describe the principles of eddy current testing. A transformer model is presented to demonstrate the fundamentals of eddy current induction and the impedance changes that occur in coil sensors. After presenting operating principles, we present a block diagram of the constituent parts of eddy current testing equipment.

### 2.1. Electromagnetic Induction and Eddy Current Inspection

Every coil is characterized by the impedance parameter *Z*_0_, which is a complex number defined as in Equation (1) and which represents the voltage-current ratio (*V*_0_/*I*_0_) for a single frequency sinusoidal excitation *f*. Impedance *Z*_0_ has a magnitude |*Z*| and a phase *φ*:

(1) $$Z_{0}=\frac{V_{0}}{I_{0}}=R_{0}+{\mathrm{jX}}_{0}=R_{0}+j2\pi fL_{0}={\sqrt{R_{0}{}^{2}+X_{0}{}^{2}}}_{\varphi =\text{atan}2(X_{0}/R_{0})}={\left|Z\right|}_{\phi }$$

When an alternating current energizes a coil, it creates a time-varying magnetic field. The magnetic lines of flux tend to be concentrated at the center of the coil. Eddy current inspection is based on Faraday’s electromagnetic induction law as demonstrated in Equation (2). Faraday discovered that a time-varying magnetic induction flux density induces currents in an electrical conductor. The electromotive force *ε* is proportional to the time-rate change of the magnetic induction flux density Φ*_B_*:

(2) $$ɛ=-\frac{d\Phi_{B}}{\mathrm{dt}}$$

**Figure 1.**
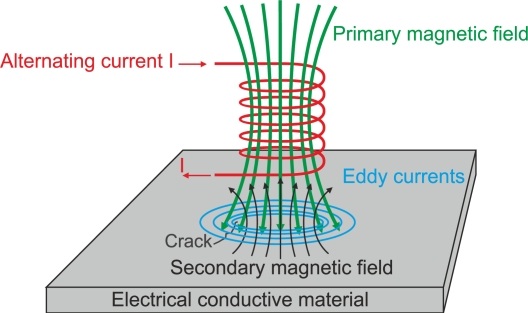
Primary and secondary magnetic field. Eddy current on the test piece (adapted from [14]).

When an alternating energized coil of impedance *Z*_0_ approaches an electrically conductive non-ferromagnetic material, the primary alternating magnetic field penetrates the material and generates continuous and circular eddy currents. The induced currents flowing within the test piece generate a secondary magnetic field that tends to oppose the primary magnetic field, as shown in Figure 1. This opposing magnetic field, coming from the conductive material, has a weakening effect on the primary magnetic field. In effect, the new imaginary part of the coil impedance decreases proportionally when the eddy current intensity in the test piece increases [12]. Eddy currents also contribute to the increasing of the power dissipation of energy that changes the real part of coil impedance. Measuring this coil impedance variation from *Z*_0_ to *Z_c_*, by monitoring either the voltage or the current signal, can reveal specific information such as conductivity and chemical composition of the test piece [13].

### 2.2. Complex Impedance Plane

This subsection describes the coil impedance changes that occur when a coil probe interacts with materials and presents the normalized impedance plane. When there is no test piece close to the coil sensor, its impedance *Z*_0_ is a complex value, as Equation (3) shows:

(3) $$Z_{0}=R_{0}+{\mathrm{jX}}_{0}$$

where *R*_0_ and *jX*_0_ are the real and the imaginary part of *Z*_0_. The component *X*_0_ = 2*πf L*_0_ is proportional to frequency *f* and the induction coefficient *L*_0_.

When a conductive test material approaches the energized coil probe, eddy currents appear on the test piece. Eddy currents create a secondary field that interacts with the primary field. As a result, the new impedance is *Z_c_* as Equation (4) demonstrates:

(4) $$Z_{c}=R_{c}+{\mathrm{jX}}_{c}$$

where *R_c_* and *jX_c_* represent the real and the imaginary parts of *Z_c_*, then *X_c_* = 2*πf L_c_* is proportional to frequency *f* and the induction coefficient *L_c_* when a test piece is close to the coil.

**Figure 2.**
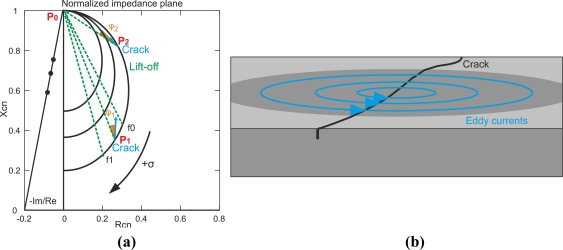
**(a)** Normalized impedance plane. Lift-off curves and crack displacement at impedance plane for two values of conductivity P1 and P2 (adapted from [12]). **(b)** Altered eddy current flow by a crack on the surface.

Coil impedance is a two-dimensional variable, and the real and imaginary parts can be represented on an impedance plane. The X-axis plots the real part of impedance, and the Y-axis represents the imaginary part. Real and imaginary impedance parts of *Z_c_* can be redefined as *R_cn_* and *X_cn_* to obtain the normalized impedance as Figure 2(a) shows [12,15]. Equation (5) indicates the transformation:

(5) $$R_{\mathrm{cn}}=\frac{R_{c}-R_{0}}{X_{0}};X_{\mathrm{cn}}=\frac{X_{c}}{X_{0}}$$

The normalized real part of the new impedance *R_cn_* is 0 when there is no change in the real part of the impedance. *R_cn_* is divided by the imaginary part of the impedance *X*_0_ when there is no metal near the sensor. *X_cn_* represents the number of times that the new imaginary part of *Z_c_* is bigger or smaller than the imaginary part when there is no target *X*_0_. To summarize, this transformation means that when there is no test piece near the coil the new impedance values become *R_cn_* = 0 and *X_cn_* = 1. This point is called “air point” *P*_0_.

#### 2.2.1. Non-ferromagnetic Material Approach

When a non-ferromagnetic material of conductivity *σ*_1_ approaches the coil probe, encircling eddy currents appear. The displacement of the normalized impedance plane is the line from the air point *P*_0_ to *P*_1_. This is the lift-off line for this material, in which conductivity is *σ*_1_. At *P*_1_ *R*_*cn*1_ > 0 as eddy currents create additional power dissipation on the test piece. However, *X*_*cn*1_ < 1, which means that *X*_*c*_ < *X*_0_. This is the effect of weakening the total field inside the coil core due to the secondary magnetic field from eddy currents.

If less conductive material (*σ*_2_) is approached, *σ*_1_ > *σ*_2_, the displacement is along another lift-off line from air point *P*_0_ to *P*_2_. Eddy current flow decreases with respect to P1. Thus, the change of resistivity of the coil is smaller than *P*_1_ as 0 < *R*_*cn*2_ < *R*_*cn*1_. The secondary magnetic field, due to eddy currents, is not as strong as *P*_1_ so that *X*_*cn*1_ < *X*_*cn*2_ < 1.

When a crack is present in the test piece, it obstructs the eddy current flow, as Figure 2(b) illustrates. There is a displacement from *P*_1_ or *P*_2_. This causes the eddy current path to become longer, and the secondary magnetic field from the eddy currents is reduced. In conclusion, the real part of impedance *R_cn+crack_*, which is related to eddy current dissipation, decreases *R_cn_* > *R_cn+crack_*, In addition to that, the sum of the primary magnetic field and secondary magnetic field increases, which means that the inductive part of impedance *X_cn+crack_* increases *X_cn_* < *X_cn+crack_*.

When approaching low conductive materials, differences between the lift-off direction and defect direction are less significant when compared to point *P*_1_; therefore, it is more difficult to distinguish between lift-off and defect indications.

#### 2.2.2. Ferromagnetic Material Approach

**Figure 3.**
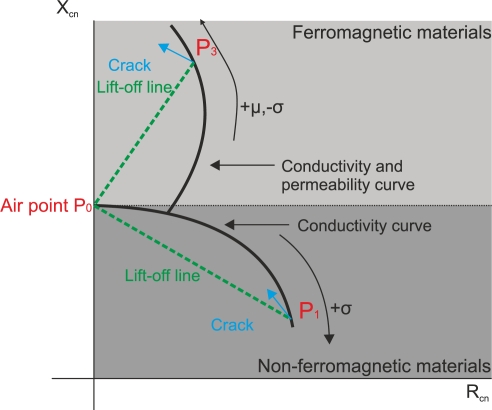
Impedance plane for ferromagnetic and non-ferromagnetic materials.

When a coil probe is in close proximity to a ferromagnetic material, such as steel or pure iron, the reactance *X_cn_* > 1 increases instead of decreases. Ferromagnetic materials, whose magnetic permeability is greater than the value of non-ferromagnetic materials, concentrate the primary magnetic field of the coil. The increase in the primary magnetic field overshadows the secondary magnetic field of the eddy currents. The displacement is from *P*_0_ to *P*_3_ and occurs in the impedance semi-plane *X_cn_* > 1 as illustrated in Figure 3.

This demonstrates that the impedance plane is divided into two semi-planes as seen in Figure 3. The normalized imaginary part of impedance *X_cn_* < 1 is the operating area of non-ferromagnetic materials. Lift-off and defects occur in this part of the plane. The normalized imaginary part of impedance *X_cn_* > 1 is the half part of the plane in which ferromagnetic materials occur.

When a crack appears, it produces the same impedance effects as non-ferromagnetic materials. A decrease in power dissipation R_cn_ > R_cn+crack_ and an increase in the imaginary part of the impedance X_cn_ < X_cn+crack_ occur.

### 2.3. Eddy Current Transformer Model

**Figure 4.**
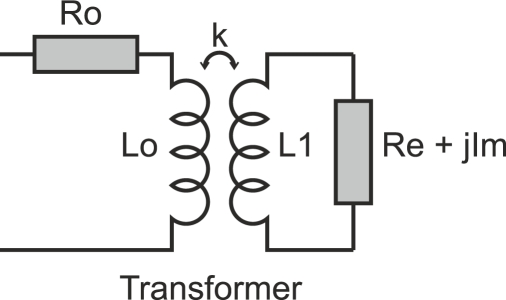
Model of coil-target interaction based on a transformer (adapted from [12]).

The transformer model of Figure 4 presents a diagram of the basic probe-flaw interaction. Some authors such as Placko *et al.* and Peng *et al.* have proposed this model to explain what occurs when the space between a coil probe and a test piece varies [12,16]. The primary circuit, whose impedance is the ratio $Z_{c}=\frac{U}{I}$, represents the coil sensor. The secondary circuit represents the test piece. The real impedance *R_e_* represents the resistance of the loops described by the flow of eddy currents. The resistor *R_e_* is consequently proportional to the resistivity of the test piece. The imaginary term *jI_m_* represents the leakage inductance of the circuit. Finally, the coupling coefficient *k* is linked to the distance between the sensor and the test piece. The coefficient *k* decreases when the distance increases.

The following Equations (6) and (7) are obtained from Kirchhoff’s Voltage Law to describe the transformer in Figure 4:

(6) $$R_{0}I+j\omega L_{0}I-j\omega M_{2}I_{e}=V$$

(7) $$R_{e}I_{e}+jI_{m}I_{e}+j\omega L_{1}I_{e}-j\omega M_{1}I=0$$

where the pulsation *ω* is related to frequency *f* as *ω* = 2*πf*, *R*_0_ and *L*_0_ are the resistance and inductance of the primary coil when no test piece is near the coil, *R_e_* and *L*_1_ are respectively the resistance and inductance of the induced eddy current loop and *M*_1_ = *kL*_0_ and *M*_2_ = *kL*_1_ are the mutual inductance between the two loops.

When there is no test piece near the coil sensor, the coupling factor *k* is zero and the measured impedance is *Z*_0_ of the primary circuit as presented in Equation (1). When a conductive test piece is approached, the complex impedance of the primary circuit becomes *Z_c_* as formulated in Equation (8):

(8) $$Z_{c}=R_{0}+{jL}_{0}\omega +\frac{k^{2}L_{0}L_{1}\omega^{2}}{R_{e}+{jL}_{1}\omega +{jI}_{m}}$$

The inductance and resistivity of the primary circuit can be identified from Equation (8) as demonstrated in Equations (9) and (10) respectively. The equivalent inductance *L* decreases due to the induced eddy currents. In contrast, the resistivity increases:

(9) $$L_{c}=L_{0}-\frac{{\left(\omega k\right)}^{2}L_{0}L_{1}(L_{1}+I_{m}/\omega )}{R_{e}{}^{2}+{\left(\omega L_{1}+I_{m}\right)}^{2}}$$

(10) $$R_{c}=R_{0}+\frac{{\left(\omega k\right)}^{2}L_{0}L_{1}R_{e}}{R_{e}{}^{2}+{\left(\omega L_{1}+I_{m}\right)}^{2}}$$

From Equation (8) the normalized real and imaginary parts of impedance *R_cn_* and *X_cn_* are presented in Equations (11) and (12) [12,16]:

(11) $$R_{\mathrm{cn}}=\frac{R_{c}-R_{0}}{X_{0}}=\frac{k^{2}L_{1}\omega R_{e}}{R_{e}{}^{2}+{\left(L_{1}\omega +I_{m}\right)}^{2}}$$

(12) $$\begin{array}{l} X_{\mathrm{cn}}=\frac{X_{c}}{X_{0}}=1-\frac{k^{2}\omega L_{1}(L_{1}\omega +I_{m})}{R_{e}{}^{2}+{\left(L_{1}\omega +I_{m}\right)}^{2}}=1-\frac{L_{1}\omega +I_{m}}{R_{e}}\frac{k^{2}L_{1}\omega R_{e}}{R_{e}{}^{2}+{\left(L_{1}\omega +I_{m}\right)}^{2}}\\ \;\;\;\;\;\;\;\;\;\;\;\;\;\;\;\;\;\;\;\;\;\;\;\;\;\;\;=1-\frac{L_{1}\omega +I_{m}}{R_{e}}R_{\mathrm{cn}} \end{array}$$

Assuming that *I_m_*, *R_e_* and *L*_1_ do not depend on the distance between the sensor and the target, the lift-off line for a fixed frequency in the normalized impedance plane can be plotted when the coupling factor *k* changes.

### 2.4. Magnetic Field Sensors for Eddy Current Testing

These non-destructive techniques need to pick up the magnetic flux from eddy currents. Many important developments have been made in magnetic sensors during the past 60 years [17]. Novkovski has researched the recent progress of state-of-the-art magnetic field sensors such as inductive coils, fluxgate magnetometers, proton precession magnetometers, superconducting quantum interference devices SQUID, Hall effect devices and magnetoresistors [17]. Nowadays, the trend in magnetometer development is toward miniaturization, and researchers are looking for new ways to reduce the size of these sensors. Section 4 reviews the most common, state-of-the-art sensors used in eddy current testing.

The magnetic field is the result of distributed currents and the distribution of ferromagnetic materials around the sensor [17]. In regions where no currents flow, the induction field is the gradient of a potential V that satisfies Laplace’s Equation (13) [18]:

(13) $$B=-\nabla V,\;\;\nabla^{2}V=0,$$

Some authors such as Backus consider a two-dimensional vector field **B** defined in some open subset V = V(z) = V(x, y) of the Euclidean plane where z = x + iy [19]. The field **B** has real and imaginary components, as Equation (14) demonstrates:

(14) B=Bx(x,y)−iBy(x,y)

Determining the real and imaginary components of **B** has several applications. For instance, by measuring the field on a grid of points, it is possible to reconstruct the currents [17]. This is an inverse problem which is solved in many non-destructive tests [17].

### 2.5. Elements of a Basic Inspection System

**Figure 5.**
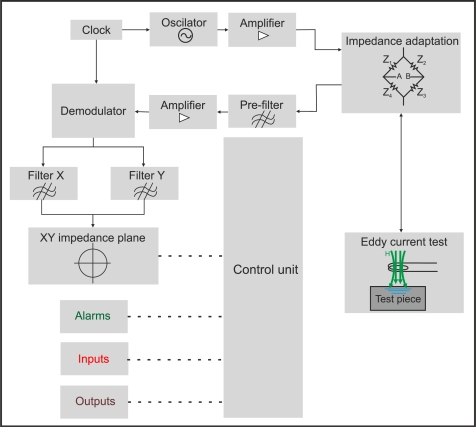
Block diagram of an analog eddy current system.

Figure 5 presents a block diagram of analog eddy current equipment. It includes a single tone generator which energizes the test coil sensor. Phase, frequency and amplitude can be adjusted to optimum parameters for the test pieces. When a crack occurs, the coil impedance experiences a change. The defect signal modulates the tone from the oscillator. A quadrature amplitude demodulator extracts the defect signal caused by the impedance variation. The demodulator outputs are X-axis and Y-axis signals. Each component represents the real and imaginary parts of the impedance respectively. These signals can be filtered and analyzed.

**Figure 6.**
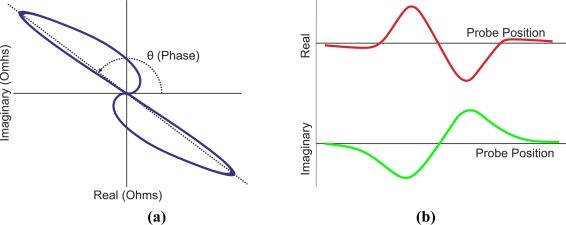
**(a)** Typical loop of a complex impedance plane of a differential probe inside a tube affected by a flaw (adapted from [13]). **(b)** Real and imaginary part of impedance change *vs.* time (adapted from [13]).

The voltage signals, which represent the impedance changes in the inspection coil, can be displayed on a XY plot. Figure 6 illustrates a typical loop of an impedance plane on a XY plot when a flawed tube is inspected using a differential coil probe. Most eddy current systems permit configuring of alarms on an XY plot to distinguish between flawed or unflawed test pieces. Alarm events can activate analog or digital outputs. In addition, modern eddy current equipment usually has digital inputs such as test piece presence or encoder connectors to start testing or to measure the speed of inspected bars or tubes respectively.

## 3. Main Variables of Eddy Current Testing

This section discusses the main variables of eddy current inspection. These variables include the electrical conductivity and the magnetic permeability of the test piece, lift-off between the coil sensor and the inspected piece, the coil fill factor for encircling probes, the edge effect, the skin effect of current distribution in the test piece, the phase lag and the signal to noise ratio. The last subsection overviews the relation between the equivalence model of eddy current sensors and their applications.

### 3.1. Electrical Conductivity of the Test piece

Materials have a characteristic resistance to the flow of electricity which is characterized with the magnitude electrical conductivity σ or its inverse resistivity $\rho =\frac{1}{\sigma }$. Conductivity is crucial in eddy current inspection.

Highly conductive materials such as cooper and aluminum create intense eddy currents and have two advantages over less conductive materials. First, cracks generate higher signal levels, as the impedance plane in Figure 2(a) illustrates. In addition to that, the phase lag between the flaws and lift-off line is larger when highly conductive materials are tested, that is *φ*_1_ > *φ*_2_ as Figure 2(a) shows. The disadvantage of highly conductive materials is that the standard penetration depth is lower at a fixed frequency than in lower conductive materials such as steel and stainless steel. Factors that exert an influence in conductivity are the temperature of the test piece, the alloy composition and the residual stress, which is related to the atomic structure.

**Figure 7.**
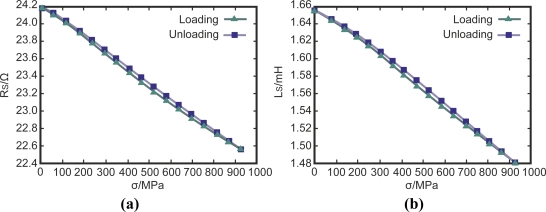
**(a)** Resistance as a function of mechanical stress (adapted from [21]). **(b)** Inductance as a function of mechanical stress (adapted from [21]).

Many authors have measured residual stress using eddy current techniques. Coils can detect very small stress variations in ferromagnetic steels due to the magneto-elastic effect [20]. Stress can be measured based on the changes in the impedance of an electromagnetic coil as Figure 7(a,b) shows [21]. The impedance change occurs due to variations in the electrical conductivity and the magnetic permeability of the test piece.

**Figure 8.**
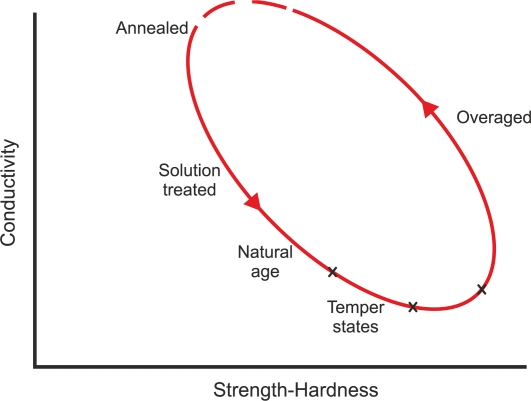
Variation of aluminum conductivity with heat treatment (adapted from [23]).

**Figure 9.**
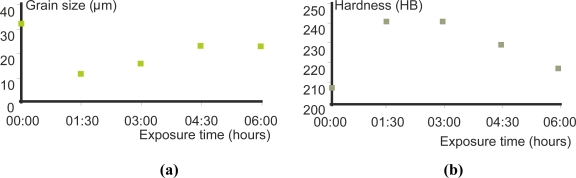
**(a)** Grain size versus exposure time, 20NC6 steel (adapted from [22]). **(b)** Hardness (Brinell) versus exposure time, 20NC6 steel (adapted from [22]).

Heat treatments cause variations of hardness, which are related to conductivity, as Figure 8 illustrates. Eddy currents can detect when pieces have received a heat treatment as well as the severity of the treatment. The eddy current testing can also characterize grain size changes after thermal treatment based on conductivity and magnetic permeability changes [22]. As Figure 9 shows, the hardness is inversely proportional to the grain size [22].

Some authors have published papers related to the conductivity of the test piece, as it is one of the most important variables in eddy current testing. Shao *et al.* presented a method for the reconstruction of conductivity profiles from eddy current impedance change data [24]. This is an inverse problem which solves the conductivity profile of the material from the electrical signal obtained in the eddy current inspection. On the other hand, other authors such as Uzal *et al.* have published numerical and analytical methods for computing the coil impedance when arbitrary radial conductivity changes occur in the test piece [25].

### 3.2. Magnetic Permeability

Magnetic permeability μ is a number that quantifies the degree of magnetic induction B of materials when a magnetic field H is applied, as shown in Equation (15):

(15) B=μH

Magnetic permeability μ is a scalar in isotropic mediums. Free space has a characteristic permeability constant *μ*_0_. In many instances, the permeability of materials is expressed as relative permeability μ*_r_* in respect of free space *μ*_0_ as Equation (16) shows:

(16) $$\mu =\mu_{r}\;\mu_{0};\;\text{where}\;\;\mu_{0}=4\pi *10^{-7}N/A^{2}$$

Materials can be classified by their magnetic properties which strongly affect the eddy current testing. The most common classification of materials depending on their magnetic response is presented below:

- Firstly, paramagnetic materials, such as aluminum, are softly attracted to magnetic fields and, hence, have a relative magnetic permeability slightly greater than one, *μ_r_* ≥ 1.
- Secondly, diamagnetic materials like copper and lead create a magnetic field in opposition to an externally applied magnetic field, thus causing a softly repulsive effect. Magnetic permeability is less than *μ*_0_; therefore, the relative permeability is a bit less than one, *μ_r_* ≤ 1.
- The third group of this classification is formed by ferromagnetic materials such as iron, nickel, cobalt and some of their alloys. These materials are strongly attracted by magnetic fields and concentrate the flux of magnetic fields. Their relative permeability is much greater than one *μ_r_* ≫ 1. One hundred or two hundred are typical values of relative permeability.

**Figure 10.**
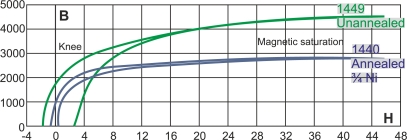
B-H curve in high nickel steel (adapted from [26]).

Figure 10 shows two magnetization curves of unannealed and annealed steel and plots the relation between B and H fields [26]. The relationship between H and B is not linear and presents hysteresis in ferromagnetic materials. The curve may be divided into two parts divided by the knee of the curve. The first part of the curve has the greater slope, and the second part has the smaller slope [27]. Saturation state is reached when the increase of H causes very little increase in B, as Figure 10 indicates.

High magnetic permeability makes the standard penetration depth decrease. In order to compensate for this effect and explore the material internally, ferromagnetic materials are inspected at lower frequencies than non-ferromagnetic ones.

Ferromagnetic materials have a characteristic property, which is a high permeability variation that presents particular difficulties when testing eddy current flow [28]. The following subsection explains this phenomenon.

#### 3.2.1. Magnetization of Ferromagnetic Materials

Large variability in magnetic permeability is a characteristic of ferromagnetic materials. This permeability variation strongly influences the eddy current flow. However, eddy current tests can also be applied to ferromagnetic materials, as the conductivity changes when a crack is close to the coil probe.

The disadvantage of inspecting magnetic materials is that permeability changes generally have a much greater effect on eddy current response than conductivity variations. This heterogeneity means that crack detection is not possible when permeability changes randomly. The equalization of the permeability is often related to how the test piece was manufactured [28]. The heterogeneity of permeability for cast iron is stronger than that of carbon steel [28].

This is a problem that many authors have taken into account. Uzal *et al.* calculated the impedance of a cylindrical air-core probe over a layered metallic material whose conductivity and permeability varied continuously as arbitrary functions of the depth [29].

The solution allowing the accuracy of the measurement of ferromagnetic materials is a process that equalizes permeability [28], such as a magnetization by means of a saturating direct-current coil. Saturated materials have a constant magnetic permeability and can be inspected with greatly reduced influence on permeability variations. The test piece must be adjacent to the magnetizer coil. The magnetization current must be sufficiently strong enough to produce magnetic saturation. Furthermore, ferromagnetic materials can be magnetized randomly due to industrial processes which present difficulties in eddy current testing. Research has been conducted in order to explore magnetizing systems for eddy current inspection. For example, Kasai *et al.* have used magnetization to cancel external magnetism [30].

### 3.3. Lift-Off

The lift-off is the impedance change that occurs when there is variation in the distance between the inspection coil probe and the test piece. The lift-off variations can be caused by varying coating thicknesses, irregular sample surfaces or the operator’s movements [31]. The magnetic field is stronger close to the coil, so lift-off is stronger near the probe. In many applications, eddy current measurements are adversely affected by lift-off [32]. Lift-off is considered a noise source and it is undesirable in defect detection. Lift-off could occur in the same direction as the crack, thereby concealing the crack response. Therefore, the distance between the probe and metal must be as constant as possible in order to avoid lift-off.

**Figure 11.**
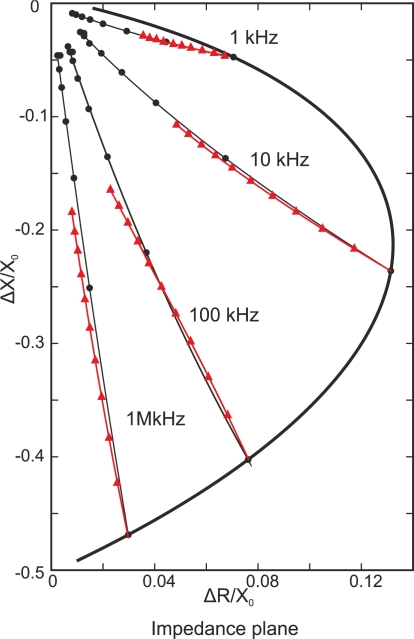
Lift-off in steps of 0.1 mm (triangle) and tilt in steps of 10° (round) for a normalized impedance plane (adapted from [33]).

At the normalized impedance plane of Figure 11, the lift-off curves start at the air point (0,0), when there is no test piece. In this case, air point is (0,0) instead of (0,1) as discussed in the previous section because a different transformation in the Y-axis has been used as shown in Equation (17). Air point corresponds to *X_c_* = *X*_0_ and therefore the normalized imaginary part is null *X_cn_* = 0:

(17) $$X_{\mathrm{cn}}=\frac{X_{c}-X_{0}}{X_{0}}$$

Figure 11 plots lift-off lines in steps of 0.1 mm. The impedance values are plotted using triangles. In some cases, when measuring the thickness of non-conductive coatings over metal, lift-off is employed as a useful property. Figure 11 demonstrates that when the test piece is closely adjacent to the coil probe, the triangle separation is larger than when the test piece is further away. This means that the resolution to measure non-conductive coatings is greater for thin coatings [33].

**Figure 12.**
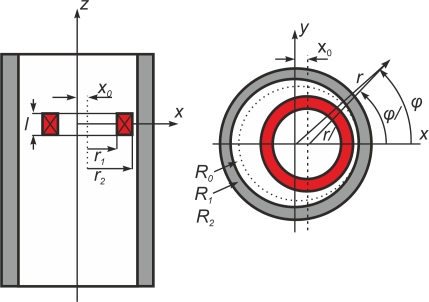
Wobble simulation: a bobbin coil in an offset position to a tube (adapted from [34]).

Lift-off is explained using a coil whose axis is normal to the test piece. However, lift-off also occurs when the test is conducted using encircling probes. The vibration of the rod or the tube inside the probe generates noise which presents difficulties in conducting inspections. Some authors including Theodoulidis *et al.* were conscious of lift-off testing tubes. They presented an analytical model of wobble in heat exchanger tube inspection with bobbin coils [34]. Figure 12 illustrates the offset position of the tube inside the bobbin coils.

There are methods for lift-off compensation when eddy currents are used in order to detect cracks and lift-off becomes an undesired variable. For instance, Yin *et al.* researched dual excitation frequencies and coil design to minimize the lift-off effect [32]. Research into processing data is also conducted, with a view to minimizing the lift-off effect. Lopez *et al.* proposed the use of wavelets to remove eddy current probe wobble noise from steam generator tubes [35]. Reduction of the lift-off effect has also been attempted by optimizing the coil design [36] and sensor array.

**Figure 13.**
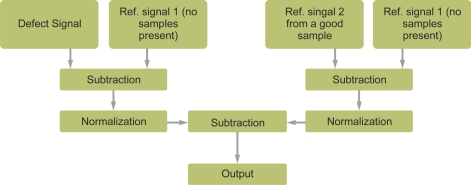
Diagram block using normalization to reduce lift-off effect (adapted from [31]).

Authors such as Gui Yun *et al.* have researched the reduction of lift-off effects via normalization techniques [31]. The technique can be applied to the measurement of metal thickness beneath non-conductive coatings and to the measurement of microstructure and strain/stress, where the output is highly sensitive to the lift-off effect. They proposed an approach using two reference signals calculated in two stages as Figure 13 shows.

The first stage was aimed to reduce the lift-off effect and used the first reference signal *y_air_* (*n*) obtained when the probe was in the air. By doing so, they created a newly derived defect signal $y_{x}^{\prime }(n)$ that was relatively free of lift-off variation as Equation (18) shows:

(18) $$y_{x}^{\prime }(n)=\frac{y_{\mathrm{air}}\;(n)-y_{x}\;(n)}{\text{max}\;\left(y_{\mathrm{air}}\;(n)-y_{x}\;(n)\right)}$$

where the defect signal is *y_x_* (*n*), *n* = [1,2,., *N*] and *N* is the number of sampled data for each signal.

The second stage was to work out the crack information. They used a second reference signal *y_ref_* (*n*), which was obtained from a good sample part. They also derived the normalized reference signal ${y^{\prime }}_{\mathrm{ref}}(n)$ as Equation (19) shows:

(19) $$y_{\mathrm{ref}}^{\prime }\;(n)=\frac{y_{\mathrm{air}}\;(n)-y_{\mathrm{ref}}\;(n)}{\text{max}\;\left(y_{\mathrm{air}}\;(n)-y_{\mathrm{ref}}\;(n)\right)}$$

Finally, a new differential signal was worked out as Equation (20) indicates. The authors obtained a significant lift-off reduction:

(20) $$d_{y}^{\prime }\;(n)=y_{x}^{\prime }\;(n)-y_{\mathrm{ref}}^{\prime }(n)$$

### 3.4. Fill Factor

Fill factor is a number which measures how well the test piece fills the coil in external encircling probes. It can be calculated as Equation (21) demonstrates:

(21) $${\mathrm{fill}}_{\mathrm{factor}}=\frac{{\left({\mathrm{Diameter}}_{\mathrm{test}\_\mathrm{piece}}\right)}^{2}}{{\left({\mathrm{Diameter}}_{\mathrm{coil}}\right)}^{2}}$$

where *Diameter_test_piece_* is the test piece diameter and *Diameter_coil_* is the diameter of the coil probe, assuming that both diameters are measured in the same units.

Fill factor is the ratio of the cross sectional area of the test piece and area of the coil section. It is necessary that the coil wires be as close as possible to the test piece, in order to have a greater response potential to cracks. In other words, it is desirable for the fill factor to be as near as possible to unity. For the internal inspection of tubes, a probe is introduced using a guidance system. The fill factor is redefined as follows in Equation (22) where it also demonstrates the desire that is nearer to one:

(22) $${\mathrm{fill}}_{\mathrm{factor}}=\frac{{\left({\mathrm{Diameter}}_{\mathrm{coil}}\right)}^{2}}{{\left({\mathrm{Diameter}}_{\mathrm{test}\_\mathrm{piece}}\right)}^{2}}$$

where *Diameter_coil_* is the outer diameter of the coil probe and *Diameter_test_piece_* is the inner diameter of the test piece, assuming that both diameters are measured in the same units.

### 3.5. Edge Effect

Edge effect is a phenomenon that occurs when an inspection coil is at the end of the test piece. In these instances, eddy current flow is distorted as currents cannot flow at the edge. So, in order to avoid the confusion with flaws, inspection is limited near edges. The distance where the edge effect is present is from approximately one to three times the diameter of the inspection coil in the case of encircling probes. So a reduction in coil size reduces the edge effect, although there is a limit, as the diameter of external encircling coils must be higher than that of the inspected materials.

Some authors have specifically addressed the edge effect in their research. For instance, Theodoulidis *et al.* proposed a model to calculate the quasi-static electromagnetic field of a cylindrical coil in the vicinity of the edge of a metal block [37]. The authors obtained some analytical expressions of fields that provided a better understanding of the edge effect and formed the basis of a procedure for solving a whole class of edge related problems.

### 3.6. Frequency and Skin Effect

Frequency inspection in eddy current testing is crucial to detecting flaws. When fixing a frequency, the initial coil impedance *Z*_0_ is adjusted. When inspection frequency *f* is increased, the imaginary part of the impedance is increased as Equation (23) demonstrates:

(23) $$Z_{0}=R_{0}+j2\pi fL_{0}$$

where *X*_0_ = 2*πf L*_0_ is the inductive reactance of the coil in ohms (Ω), *f* is the test frequency in Hertz (Hz) and *L*_0_ is the inductance in Henrys (H).

Eddy current flow is not uniformly distributed throughout the entire volume of test pieces. Current flow is stronger at the surface, decreasing exponentially by increments in relation to the distance from the surface. Assuming that the current density flowing along X axis, Equation (24) represents this current flux:

(24) $$\vec{J}=J_{x}(z,t)*\vec{u_{x}}$$

where $\vec{u_{x}}$ the unitary vector along X axis and *J_x_* (*z*, *t*) is the magnitude of density current as function of depth *z* and time *t*. Equation (25) shows the phasor of the current density along depth (Z axis) [38]:

(25) $$J_{x}(z)=J_{0,\max}e^{-\frac{z}{\delta }}e^{j\left(\alpha_{0}-\frac{z}{\delta }\right)}$$

where *J*_0,*max*_ is the maximum current density at surface and *z* is depth. The standard penetration depth *δ* is the depth at which the eddy-current density decreases to a level of about 37% of its surface value. The term *α*_0_ is the phase at *t* = 0 and *z* = 0 and $-\frac{z}{\delta }$ is the phase lag. Equation (26) demonstrates the current density as a real signal [38]. This equation is extracted from Equation (25) taking the real part. It reveals that the current density phase varies 1 radian when the distance traveled from the surface is *δ*:

(26) $$J_{x}(z,t)=\mathrm{Real}(J_{x}(z)*e^{j\omega t})=J_{0,\max}e^{-\frac{z}{\delta }}\text{cos}\left(\omega t+\alpha_{0}-\frac{z}{\delta }\right)$$

**Figure 14.**
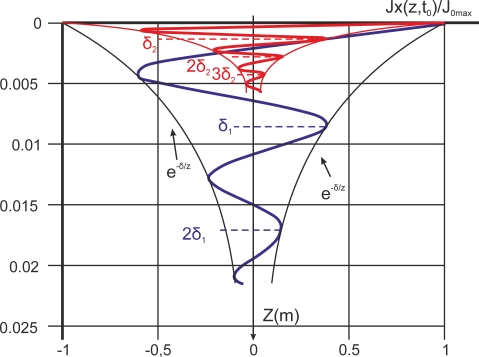
Electromagnetic field penetration inside pure aluminum at frequencies of 200 Hz and 10 KHz (adapted from [38]).

Standard penetration depth depends on electrical conductivity, the magnetic permeability of the test material and on the eddy current frequency. Standard penetration depth is lower as conductivity, permeability or inspection frequency increase. The penetration depth can be calculated as Equation (27) expresses [38]:

(27) $$\delta =\sqrt{\frac{2}{\mu \omega \sigma }}$$

where *δ* is the standard depth of penetration in meters, *σ* is the conductivity in 1/(Ω*m*), *μ* is the magnetic permeability *μ* = *μ_r_μ*_0_ and *ω* = 2*πf*. The testing frequency *f* is in Hz. Resistivity *ρ* is the reciprocal of conductivity *ρ* = 1/*σ*. As an example, Figure 14 illustrates the electromagnetic field penetration inside aluminum at two different frequencies (200 Hz and 10 kHz) [38]. Typical values of standard penetration depth for pure aluminum are 5.99 mm at 200 Hz and 0.847 mm at 10 KHz.

Equation (27) demonstrates that low frequency tests increase the standard depth of penetration and are more suitable for inspecting subsurface flaws. Some authors have researched the detection of subsurface defects, including Ramos *et al.* regarding the characterization of depth profiles of subsurface defects in aluminum plates [38].

Skin effect is also a limiting factor of increasing frequency as desired. The thickness of the inspected material must be two or three times the standard depth of penetration to prevent the eddy current flow from appearing on the other side of the test piece.

Typical inspection frequencies in eddy current testing are in the range of 100 Hz–10 MHz. Most authors such as Ditchburn *et al.* [39] and Thollon *et al.* [15] use this range. However, a few authors such as Owston use higher frequencies. Owston described a high frequency eddy-current apparatus working at 25 MHz for detecting surface defects and thin metallic coatings [40].

Low frequency tests are commonly used in the inspection of ferromagnetic materials to compensate for their high permeability and penetrate into the test piece. On the other hand, the inspection of small discontinuities occurring in the near-surface region is recommended at high frequency to maximize eddy current flow at the surface.

Skin effect and other parameters such as the crack morphology and crack position with respect to the surface determine a band of operating frequencies where the cracks are detectable. At the optimum frequency of testing, the crack sensitivity reaches the maximum.

#### 3.6.1. Multi-frequency Techniques

Multi-frequency techniques are widely used in non-destructive eddy current testing. Multi-frequency testing operates at two or more test frequencies. Multi-frequency techniques expand the capabilities of single-frequency testing and save time since they allow simultaneous tests. Multi-frequency testing is also applied to cancel out undesired signals in order to improve the signal-to noise ratio [41].

The multi-frequency process uses a composite signal and subtracts the undesirable signal. Noise sources that can be minimized are probe lift-off, temperature variation, and geometrical changes in the material [41].

Multi-frequency techniques are usually accomplished by combining the results obtained at different frequencies in the spatial domain. For instance, the authors Liu *et al.* proposed a pyramid fusion method to integrate two-dimensional spatial domain with multi-frequency injection [41]. A signal-to-noise ratio criterion was adopted to evaluate the fusion results which demonstrated the potential of signal enhancement via fusion strategy.

Other authors combined raster scanning and multi-frequency techniques. Raster scanning produces images of the impedance or impedance changes over a two-dimensional (2-D) surface. These acquired images are complex values because the impedance produces complex data. Image processing techniques can be applied to detect cracks using eddy current testing. Bartels *et al.* have proposed a multi-frequency eddy current image processing technique for the non-destructive evaluation of materials [42]. 2-D eddy current testing generated a sequence of complex valued images which were linearly combined to maximize the signal-to-noise ratio SNR of features of interest. This technique consisted of a selection of weights for a linear combination of the images as shown in Equation (28) [42]:

(28) $$d(x,y)=\sum_{i=1}^{{2N}_{f}}c_{i}f_{i}(x,y)$$

where *d*(*x*, *y*) is the linear combination of images, *N_f_* is the number of test frequencies and *f*1 = *real*(*h*1), *f*2 = *imag*(*h*1), *f*3 = *real*(*h*2), *f*4 = *imag*(*h*2) … are extracted from the 2-D images *h*1, *h*2 … *hN_f_*. Results on experimental data demonstrate SNR improvement up to 1100 percent over traditional two-frequency techniques.

#### 3.6.2. Pulsed Eddy Current Testing

Conventional eddy-current equipment employs a single sinusoidal excitation. These systems are strongly limited by the depth of penetration of eddy currents. Therefore, conventional systems are useful for detecting surface and near-surface cracks up to a depth of a few millimeters below the surface [43]. A solution to increase the subsurface testing is to reduce the operational frequency in order to increase the standard skin depth. However, in many cases the signal-to-noise ratio is reduced, as Faraday’s voltage law states that the induced voltage in coil sensors is proportional to the rate of change of the magnetic field.

In contrast to the conventional eddy-current instrument, pulsed instruments generate square, triangular or a saw tooth waveform [44]. These waveforms have a broad spectrum of frequencies; hence, pulsed eddy current testing techniques provide more information than traditional eddy current testing methods that can be used for the detection and characterization of hidden corrosion and cracking [45]. The data at different frequencies can be correlated to obtain the defect depth.

Pulsed eddy current instruments are classically implemented with one double-function coil or two separate coils formed by a transmitter and a receiver coil. Some authors such as Dolabdjian *et al.* employed a high-performance giant magnetoresistance magnetometer instead of the receiver coil [46].

Pulsed eddy current systems rival single or multifrequency testing, since the advantage of a transient system is that the response contains as much information as an entire spectrum of frequency-domain excitations [46]. The performance of defect classification using the pulsed technique is better than the conventional method [47].

Pulsed eddy current is useful for more than just crack detection. Haan *et al.* have used pulsed eddy current to accurately characterize the permeability and the conductivity [48, 49]. Taking a reference measurement of an object with a known thickness, they also determined the thickness of several types of carbon steel materials, which was proportional to the product of conductivity and magnetic permeability.

Typical features such as peak amplitude and zero-crossing time of pulses are employed to detect and characterize defects [50]. A Hilbert transform can also be computed to extract a new descending point feature of the received pulses [51].

**Figure 15.**
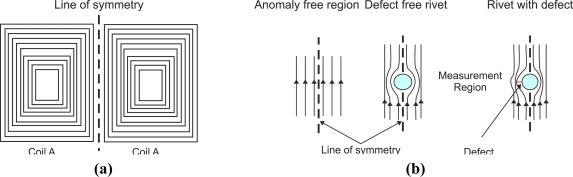
**(a)** Schematic of the multi-line coil for inducing linear eddy currents (adapted from [43]). **(b)** Induced eddy current flow in the absence and presence of rivet and cracked rivet (adapted from [43]).

Some authors have conducted research into pulsed eddy-current techniques. Many years ago, in 1969, Waidelich *et al.* researched the attenuation of a pulsed field by a conducting sheet [52]. They investigated how to increase the spatial resolution by putting the coil probe in a copper enclosure with a small aperture. Other authors such as Guang *et al.* presented a system for the inspection of aircraft structures [43]. The system generated pulse excitation that energized a planar multi-line coil of Figure 15(a). The transient field was detected via a giant magnetoresistive GMR field sensor placed on the line of symmetry at the center of the source coil. In the absence of discontinuities, the normal component of the magnetic field was zero at the center of the source coil. When the uniform distribution of the induced currents was distorted by a rivet and/or crack as sketched qualitatively in Figure 15(b) the zero field on the line of symmetry was destroyed and a nonzero transient signal of the normal component was measured by the GMR sensor.

**Figure 16.**
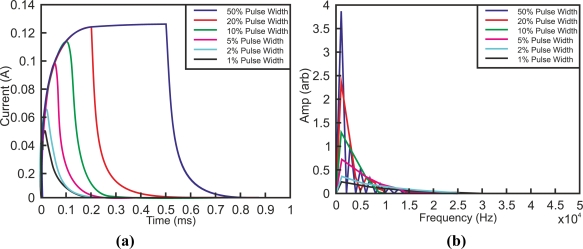
**(a)** Excitation current input with varied pulse width (adapted from [53]). **(b)** Spectrum distribution under different pulse widths (adapted from [53]).

Other researchers such as Abidin *et al.* studied the influence of duty cycle in pulses testing rivet joints [53]. Figure 16(a) shows different pulse width excitations, and Figure 16(b) shows spectrum distribution. Wider pulses are richer in low-frequency components compared to narrower pulses that are dominated by high-frequency components.

### 3.7. The Phase Lag

The phase lag is the parameter that permits the user to obtain information regarding the depth of a defect within a material. The phase lag is represented by the term $\varphi =-\frac{z}{\delta }$ in Equation (29) [38].

The phase lag represents the shift in phase between the defects on the surface and defects at *z* distance from the surface:

(29) $$J_{x}\;(z,t)=J_{0,\max}\;e^{-\frac{z}{\delta }}\;\text{cos}\;\left(\omega t+\alpha_{0}-\frac{z}{\delta }\right)$$

The phase lag depends linearly with depth *z*. When the defect is at one standard depth of penetration *z* = *δ*, the phase lag *φ* is *φ* = 1 *radian* ≈ 57°. When it is at two standard depths of penetration *z* = 2*δ*, the lag occurs at *φ* = 2*radian* ≈ 114° with respect to surface cracks. As a result, the phase lag can be used to determine the depth of subsurface defects. Using the complex impedance plane, the lift-off line can be taken as a reference phase as it occurs on the surface. Flaw direction can be measured with respect to the lift-off phase. It is desirable to have phase resolution between the lift-off line and cracks.

### 3.8. Signal-to-Noise Ratio

The signal-to-noise ratio (SNR) is a parameter that quantifies the number of times that the signal amplitude from the response to a crack is greater than the signal amplitude of the background noise. Noise sources limit eddy current testing. Some of the main noise sources in eddy current testing are temperature variations, lift-off, changes in the electromagnetic properties of the material such as conductivity or magnetic permeability and changes in test speed. Some methods for maximizing the SNR are listed below.

The simplest way to increase the SNR is to amplify the signal level. However, amplifiers increase the noise level and introduce their own noise. Therefore, there is a limit to the number of amplification stages that can be applied.

Another way to minimize noise is filtering. Filtering is possible if the perturbation is not in the pass band of the desired signal. Also, if there is phase difference between defects and the noise source, then phase discrimination techniques can be applied.

In addition, some types of coil probes are less influenced than others by some noise sources. For instance, self-compensated differential coil probes are less sensitive to small variations in diameter, conductivity or magnetic permeability than absolute coil probes. In some instances, copper shields cover the probes to decrease the pick-up noise from external sources; therefore, they increase the signal to noise ratio.

Coil size is also crucial in order to obtain a high-level signal for crack detection. It is crucial that the fill-factor is close to one in the case of encircling coil probes, and it is also crucial that the coil size is similar to the crack size. Some authors such as Grimberg *et al.* [54] take the coil size into account.

Another technique used to maximize the SNR is magnetization. As explained in the previous section, direct current magnetization minimizes the effect of permeability variations in ferromagnetic materials.

The last proposed method to improve the SNR is the selection of the most suitable sensor, as every sensor has limitations in sensitivity and noise level. In some applications, the magnetic field levels are so low that standard coil probes cannot be used to detect them. In these instances special magnetometers such as superconducting quantum interference devices (SQUID) are sensitive to extremely low field levels. SQUIDs have been used in eddy current testing for 30 years [55]. However, the disadvantage of SQUIDs is that they require a cryostat to maintain them at very low temperatures.

### 3.9. Equivalence Model of Eddy Current Coil Sensors for Applications

This subsection presents a review of the equivalence model of eddy current sensors and its relation to applications. Eddy current testing uses the electromagnetic properties of materials that depend on their composition, microstructure and the applied and residual stresses [22]. These properties are measured via the impedance Z_c_ described in Section 2, which is a function of lift-off, target conductivity σ, target magnetic permeability μ and the eddy current frequency f as Equation (31) shows:

(30) $$Z_{c}=R_{c}+{\text{jX}}_{c}=\text{function}\;(\text{lift}-\text{off},\sigma ,\mu ,f)$$

Some authors such as Tian *et al.* have researched the influence of the heterogeneity of the test piece in eddy current sensors [28]. When measuring one of these variables, such as lift-off, in Equation (30), conductivity σ and permeability variations of the test piece are noise sources that influence the test. When frequency f is high enough, the approximation shown in Equation (31) can be done [28]:

(31) $$L_{c}=L_{0}-\frac{{\left(\omega k\right)}^{2}L_{0}L_{1}(L_{1}+I_{m}/\omega )}{R_{e}{}^{2}+{\left({\omega L}_{1}+I_{m}\right)}^{2}}\approx L_{0}-\frac{M^{2}}{L_{1}}$$

Increasing the excitation frequency can suppress the influence of the non-equalization of the conductivity of the test piece R_e_ as R_e_ ≪ ωL_1_. The heterogeneity in non-ferromagnetic metals such as aluminum and copper due to conductivity variations is much lower than that in ferromagnetic metals, since the conductivity for aluminum and copper is much smaller than those of steel and cast iron which allow the approximation of R_e_ ≪ ωL_1_ to be more true.

The effect of magnetic permeability heterogeneity in non-ferromagnetic targets is much less than the heterogeneity of ferromagnetic targets. The measuring accuracy of non-ferromagnetic targets can be higher than that of ferromagnetic targets.

With regard to microstructure, Mercier *et al.* used eddy currents to evaluate steel decarburizing in the austenitization process [8]. Decarburizing can change the microstructure and the mechanical properties of steel. Changes in electrical conductivity and magnetic permeability occur in the decarburized surface.

Zergoug *et al.* analyzed the relation between mechanical micro-hardness and impedance variations in eddy current testing [22]. The characterization of the microstructure modifications due to heat treatment and corrosion by eddy currents permitted the measuring of mechanical and metallurgical parameters of materials.

In ferromagnetic materials, the use of a low frequency provides a good impedance resolution. The most significant result in the case of ferromagnetic materials characterization is the relationship between the electric and magnetic parameters and the hardness and the grain size. The hardness is inversely proportional to the grain size.

Schoenekess *et al.* detected tensile stress alterations in prestressing steel using eddy current testing [56]. Changes in mechanical stress shift the electrical conductivity and magnetic permeability of the material and are always very small, typically less than 1% [57]. Temperature compensation of the entire measurement system was absolutely necessary to minimize measurement errors.

## 4. Sensors

There are many types of magnetic sensors for non-destructive evaluation such as solenoid coil probes, superconducting quantum interference devices (SQUIDs) and Hall-effect and magnetoresistive sensors. This section presents these types of sensors and includes the most recent research of authors in sensor design.

### 4.1. Coil Probes

Coil probes are the most widely used sensors in eddy current inspection. This subsection presents a discussion regarding different coil probe types, the most important parameters in coil probes and the circuitry used to pick up signals.

#### 4.1.1. Coil Probe Types

Different coil probe structures are available to detect a large variety of cracks. In general, coil probes provide high crack sensitivity when eddy current flow is strongly altered by discontinuities.

##### Encircling Coil Probes

**Figure 17.**
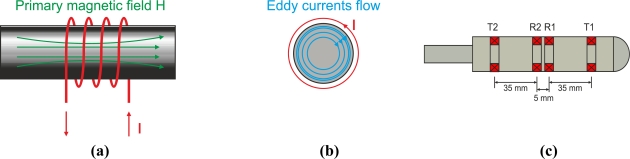
**(a)** External encircling-type coil for tube or bar inspection. **(b)** Eddy currents flow in an external encircling-type coil. **(c)** Internal encircling-type coil for tube inspection (adapted from [58]).

The most widely-used probes encircle the test piece in eddy current testing. These probes are commonly used to test bars or tubes either externally or internally and are shown in Figures 17(a,c). Encircling coils are sensitive to parallel discontinuities to the axis of the tube or bar as eddy currents describe radial circumferences in an opposing sense of currents around the energized coil current, as shown in Figure 17(b). Internal encircling coil probes permit internal testing of tubes. These types of probes are introduced using a guidance system which incorporates an encoder to locate the cracks by measuring the distance from the tube edge to the defect. Internal encircling probes usually test heat exchanger tubing at power plants at a constant rate of speed. Figure 17(c) shows an internal coil probe for ferromagnetic inspection [58].

The standard section of encircling probes is circular. In addition to that, special profile encircling probes are designed for researchers and manufacturers to control surface and sub-surface defects in products with special profiles and shapes [59].

##### Pancake-Type Probes

Pancake-type probes are coils whose axis is perpendicular to the surface of the test piece. Pancake probes can be either air-core coils or ferrite-core coils. Ferrites have high permeability and the initial coil impedance is higher than the permeability of air-core coils. Pancake-type probes are very sensitive to lift-off and inclination with respect to the flat surface. Theodoulidis evaluated the influence of tilted coils in eddy current testing [33].

**Figure 18.**
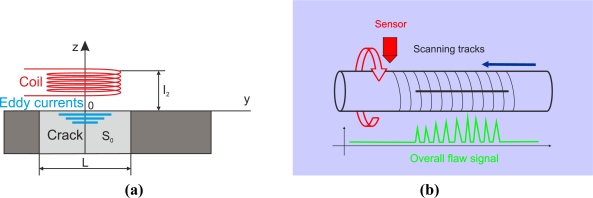
**(a)** Pancake-type coil probe and eddy current flow (adapted from [24]). **(b)** Rotating eddy current testing (adapted from [60]).

These types of sensors are used in flat surface inspection. The eddy currents on the test piece are circumferences parallel to the surface as Figure 18(a) illustrates. When a penetrating crack occurs on the surface, current flow is strongly altered and the crack can be detected. Pancake-type coil probes are not suitable for detecting laminar flaws as currents flow parallel to the surface and they are not strongly distorted.

Pancake-type probes can be used in either manual or automatic eddy current testing. Manual probes are designed especially for testing the surface defects of parts that require supervision and are particularly suitable for the maintenance of aeronautic parts. Pancake-type probes can also automatically detect longitudinal cracks in tubes or bars using a rotating system. The eddy current probe rotates at a high speed around the test material, which is moved longitudinally, and scans its surface helically as Figure 18(b) illustrates [60].

##### Other Eddy Current Probes

Other probes that are used in eddy current testing are segment probes, horseshoe-shaped coil probes, spiral coil probes and coil probe arrays.

Segment probes are used for the detection and control of defects in the weld seam of welded pipes [59]. These probes are available with specific windings and can inspect the tube or bar in differential and absolute modes. Both modes can be implemented in the same probe. In differential mode, the sensor is highly sensitive to punctual defects in the weld seam. Differential segment probes present difficulties detecting long defects in the weld seam of tubes and in the absence of a seam. Differential segment probes only detect the beginning and the end of the crack. To compensate for this disadvantage, absolute mode probes are incorporated along with differential ones to detect the presence or absence of weld seams and long cracks.

**Figure 19.**
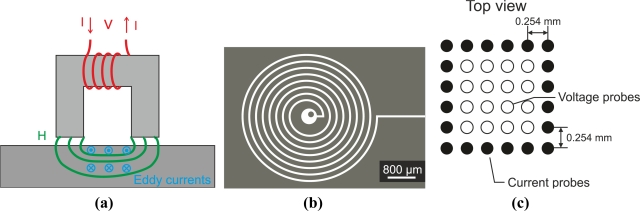
**(a)** Horseshoe-shaped coil probe (adapted from [12]). **(b)** Drawing of a 10-turn circular spiral coil (adapted from [39]). **(c)** Coil matrix (adapted from [61]).

Figure 19(a) shows a horseshoe-shaped coil, which is useful in the detection of laminar flaws. The authors Placko *et al.* used this type of probe to inspect graphite composite materials [12]. The magnetic flux penetrates parallel to the surface, and the eddy currents encircle the magnetic flux lines in the test piece as Figure 19 (a) shows. Laminar flaws alter eddy current flow significantly, which explains their high sensitivity to them.

Some authors have tested spiral coils in eddy current testing. Ditchburn *et al.,* for instance, presented the detection of long cracks in steel using the probe shown in Figure 19(b) [39]. Eddy currents describe circumferences on the test piece surface. The authors asserted that spiral coils offered attractive features in terms of sensitivity. Arrays of coils create electromagnetic eyes used in eddy current testing as Figure 19(c) illustrates. Coil matrices permit 2D image extraction and the use of image processing techniques. The space resolution depends on the coil size and can be increased via miniaturization as Zaoui *et al.* published [62]. Other authors such as Stander *et al.* used matrix coils to test green-state metal powder compacts [61].

#### 4.1.2. Double-function Probes *vs*. Separate-function Probes

This subsection presents two types of probes: double-function and separate-function probes.

**Figure 20.**
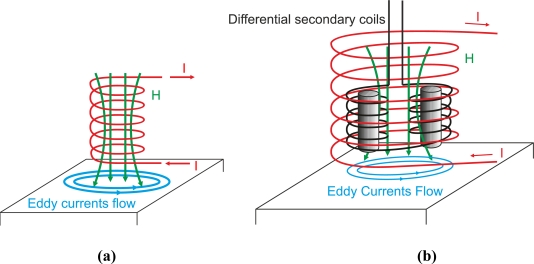
**(a)** Double-function single coil probe. **(b)** Differential separated function probe.

On the one hand, double-function probes, also called reflection probes, use the same coil or the same coils to generate eddy current flow in the test piece and to receive the secondary field from the eddy currents. Figure 20(a) shows a double-function probe formed by a single coil.

On the other hand, separate-function probes do not use the same coils to generate eddy current and to pick up the secondary field as Figure 20(b) shows. The primary coil can be specially designed to create eddy current flow. Secondary coils are made small to receive the secondary field from eddy currents with enough sensitivity [63]. The advantage of separate-function probes is that the coil design can be optimized. Primary coil impedance can be adjusted to produce a strong and uniform primary magnetic field by adjusting parameters such as coil diameter, wire diameter and number of turns. Secondary coils can be designed to pick up the maximum secondary field by minimizing noise sources and adapting the coil size to the crack size. Four combinations can be created as double or separate-function probes, which can be absolute or differential. The following subsection permits a better understanding of these configurations.

#### 4.1.3. Absolute-Mode Probe

**Figure 21.**
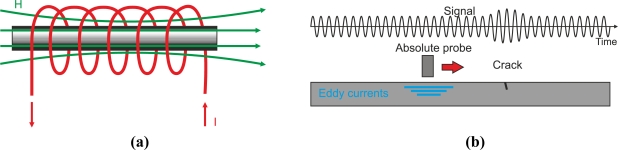
**(a)** Non-compensated absolute encircling coil probe. **(b)** Absolute signal from non-compensated absolute encircling coil probe when a cracked bar is tested.

The simplest absolute probes consist of a single coil that generates eddy currents and senses changes from the eddy current field as Figure 21(a) shows. Absolute probes provide an absolute voltage signal as Figure 21(b) illustrates. The disadvantage of these coil probes is their high sensitivity to temperature variations.

**Figure 22.**
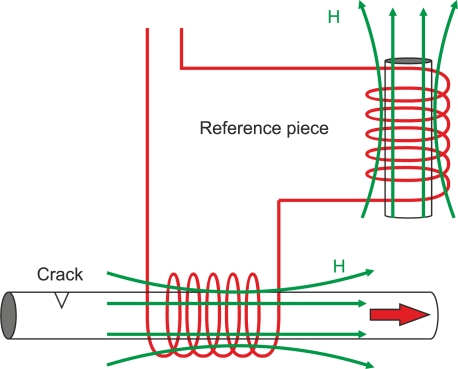
Compensated absolute encircling coil probe.

Absolute-mode probes may have a voltage compensation using an additional reference coil that is far from the inspected material as Figure 22 illustrates. A null voltage signal is measured when there is no defect which increases the instrument’s dynamic range. Furthermore, they are less sensitive to temperature changes than non-compensated probes.

Absolute probes detect long flaws or slow dimensional variations in tubes or bars, which differential probes cannot detect. In addition to crack detection, the absolute change in impedance of the coil probe provides much information about the test material such as grain size, hardness and stress measurement.

#### 4.1.4. Differential-Mode Probe

**Figure 23.**
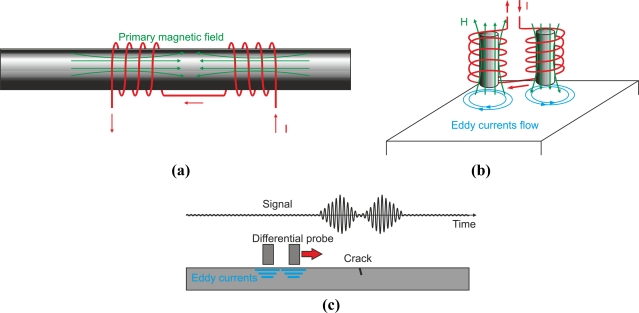
**(a)** Differential double-function encircling coil. **(b)** Differential double-function pancake-type coil. **(c)** Signal from differential coil probe.

Differential probes consist of two coils that compare two adjacent parts of the inspected material as Figure 23(a) and Figure 23(b) show. The detecting coils are wound in the opposite directions to one another in order to equalize the induced voltages originated by the excitation primary field as Figure 23(a) illustrates [63]. The output voltage of the differential coil probe is zero when there is no crack inside the probe as Figure 23(c) illustrates [6]. Cracks in the test material, which moves at a constant speed, alter the balance, and two pulses in the voltage signal are detected as Figure 23(c) shows.

Differential coils have the advantage of being able to detect very small discontinuities. However, differential coils do no detect gradual dimensional or composition variations of the test piece, as the coils are typically very close.

Many authors have attempted to improve differential coil probes in terms of crack sensitivity. Peng *et al.*, for instance, presented a new differential sensor composed of double gradient winding coils [16]. Others like Bae *et al.* used a differential probe in hot wire testing [6].

#### 4.1.5. Crack-Probe Interaction Models

Many authors have researched models of crack-probe interaction that contribute to the development of optimized probes. These scientists typically distinguish between forward and inverse solutions for the probe-crack problem.

On the one hand, the forward solution consists in predicting the impedance or voltage of the eddy-current probe coil when the cracked piece is tested by a probe [64]. Some authors have published models for obtaining the forward solution. For instance, Skarlatos *et al.* presented a model to solve the forward problem in cracked ferromagnetic metal tubes [58]. Others like La *et al.* proposed a parametric model to estimate the impedance change caused by a flaw using the electromagnetic quasi-static approach [64]. Bowler *et al.* solved the harmonic functions of the Laplace equation to calculate the impedance change of the excitation coil inspecting aluminum and steel [65].

On the other hand, the inverse solution determines the type and size of cracks from the electric signal of eddy currents. Some authors have published papers solving the inverse problem. For example, Uzal *et al.* used a recursive Bayesian estimation method to extract the properties of the test piece [25], and Tamburrino *et al.* applied communications theory [66].

#### 4.1.6. Conventional and Transmission Eddy Current Method

**Figure 24.**
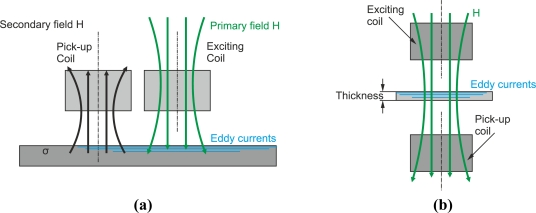
**(a)** Conventional eddy current method (adapted from [32]). **(b)** Transmission eddy current method.

Sometimes authors use the terms conventional and transmission method. The conventional method, which is the most widely used, consists of positioning the exciting and pick-up coils in the same side of the inspected material as Figure 24(a) shows [32]. The transmission method is for separate-function probes and consists in positioning the pick-up coil on the other side of the magnetic source as Figure 24(b) illustrates. The transmission method needs a maximum thickness of the test material of 3–5 times the standard penetration depth to be able to receive the signal in the pick-up coil.

#### 4.1.7. Coil Probe Circuitry

**Figure 25.**
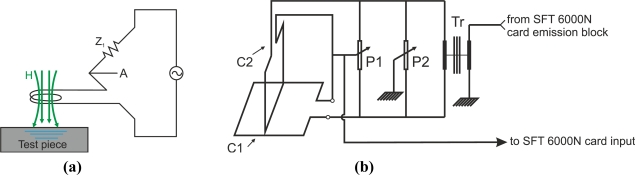
**(a)** Resistor-coil probe polarization. **(b)** Separate-function coil probe polarization (adapted from [54]).

This subsection describes how to energize coil probes for eddy current testing. The simplest method for connecting an absolute coil probe is to use the RL circuit to measure the voltage *V_A_*, as Figure 25(a) illustrates, although this configuration has the disadvantage of being sensitive to temperature changes.

**Figure 26.**
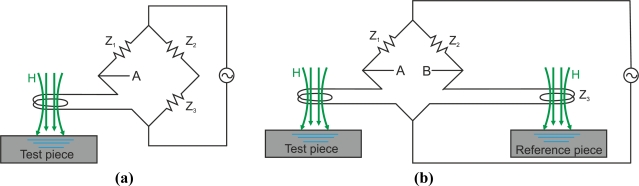
**(a)** Unbalanced bridge connection. **(b)** Balanced bridge connection.

The most widely used circuitry for eddy current coil sensors is the bridge mode, which can be balanced or unbalanced depending on the probe type. Non-compensated absolute coil probes can be polarized in serial connection with a resistor in one leg, as Figure 26(a) shows, and a balancing impedance network formed by *Z*_1_ and *Z*_2_ in the other leg. The voltage differences are measured between the two legs *V_AB_*. The balancing network permits the use of the entire range of the instrument with respect the single RL circuit. The disadvantage of this configuration is that it is also not compensated with regard to temperature, as the coil probe and impedances *Z*_1_ and *Z*_2_ have different temperature coefficients.

Compensated absolute coil probes can be polarized in both legs of the bridge in order to balance it as Figure 26(b) illustrates. The system has the advantage of being temperature compensated.

The circuitry for separate-function differential probes is commonly done by connecting the primary circuit using an RL circuit. The secondary pick-up coils may be connected directly to the input of a differential amplifier.

Not many authors have published on the coil connection. However, Grimberg *et al.* explained how they energized the coils as Figure 25(b) illustrates [54]. These coils were fed by a magnetic transformer, and the voltage was picked up by the card input and was regulated by means of the potentiometer P1.

### 4.2. Magnetoresistive Sensors

Magnetoresistive sensors are magnetic field transducers that exhibit a linear change in electrical resistance under an external magnetic field [67]. Magnetoresistive sensors are highly sensitive and accurate, but the main disadvantage of them is the high temperature coefficient [17]. Germano *et al.* presented transference curves for two types of magnetoresistive sensors: spin-valve (SV) and magnetic tunnel junction (MTJ) sensors [67].

**Figure 27.**
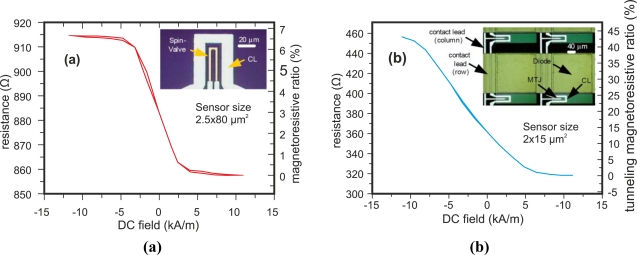
Microphotography and transfer curve of two types of magnetoresistive sensors: **(a)** Spin-valve in a linear array (adapted from [67]). **(b)** Magnetic tunnel junction in a matrix (adapted from [67]).

SV magnetometers are spin-valve transistors used as magnetic field sensors and have a ferromagnet–semiconductor hybrid structure [17]. The magnetic tunnel junctions are based on a spin dependent tunneling effect [17]. Two transfer curves of these magnetoresistive sensors are shown in Figure 27, which demonstrates that resistance decreases when the field strength increases.

Magnetoresistive sensors can be used in non-destructive evaluation to detect the secondary field from eddy currents. Some researchers such as Ramos *et al.* [38]. and Yamada *et al.* [68] use these types of sensors Yamada *et al.* used an SV-GMR sensor whose operating range of magnetic field density was from nT to mT. The sensor provided high sensitivity over frequencies up to 100 MHz and high spatial resolution due to the minizaturation [68].

### 4.3. Hall-effect Sensors

**Figure 28.**
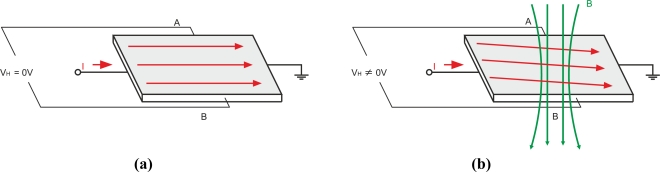
Hall-effect principle. **(a)** No magnetic field. **(b)** Magnetic field applied.

Hall-effect sensors can detect magnetic fields from eddy currents and can be used in eddy current testing. Hall voltage is proportional to the current flowing through the conductive rectangle and the magnetic induction perpendicular to the conductor as Figure 28 shows. The Hall devices are used mainly in the mT range and can be easily miniaturized and integrated within microelectronic circuits [17]. Their disadvantages are their limited sensitivity to silicon, the high level of 1/f noise and the relatively large offset [17].

Some authors such as Jongwoo *et al* have researched eddy current testing using Hall-effect sensors. They presented a quantitative eddy current evaluation of cracks on austenite stainless steel using a Hall-effect sensor array [69].

Other researchers such as He *et al.* tested the use of a differential hall probe to detect defects in the riveted structures of aircrafts [50]. The hall response signals were disturbed by noise, which leads to inaccuracy in detecting the defects. They used an averaging method and wavelet de-noise method to process the Hall responses [50].

Paasi *et al.* presented a three-axis Hall sensor magnetometer for the testing of superconductor homogeneity to measure the three components of the magnetic fields from eddy current flow [70]. The three-axis Hall sensor provides increased sensitivity when compared to classical Hall sensor techniques that measuring only one (usually vertical) component of the field.

### 4.4. SQUID Devices

Superconducting quantum interference devices (SQUIDs) are very sensitive magnetometers designed to measure extremely weak magnetic fields. SQUIDs are based on superconducting loops that contain Josephson junctions [17]. SQUIDs are sensors that can measure extremely low magnetic induction levels. The disadvantage of these types of sensors is the need for cryogenic refrigeration in order to decrease noise levels to the range of $\text{fT}/\sqrt{\text{Hz}}$ that limit their use in many applications.

SQUIDs have been used in eddy current testing since the 1980s [55]. In conventional eddy-current systems, where the magnetic field produced by the eddy currents is detected by means of an induction coil, the typical field noise is about $1\;\text{nT}/\sqrt{\text{Hz}}$ at eddy current frequencies of about 100 kHz. In some cases, this field noise is too high for certain applications such as the detection of tiny oxide particles, especially if the test materials are highly conductive, such as copper or aluminum. In these instances, SQUID magnetometers must be used instead of coil probes.

Some authors have researched eddy current testing using SQUIDs. For instance, Muck *et al.* tested various materials and obtained a much higher sensitivity than conventional eddy current evaluation and ultrasonic testing [71]. Others such as Ruosi *et al.* presented their experimental and numerical detection results of surface and subsurface artificial features in Al-Ti planar structures [72].

The combination of high sensitivity, even in unshielded environments, high spatial resolution and flat frequency response up to 1 MHz offered by SQUIDs mean that they are powerful sensors for eddy current evaluation [72].

### 4.5. Comparison of Different Probe Structure and Magnetic Sensors

There are some parameters, including the magnetic field range, the operating frequency band and sensor dimensions that permit the selection of the most suitable sensor type for eddy current testing [68]. In this subsection, a comparison of different probe structures and magnetic sensors is presented.

Coil probes provide high sensitivity to defects when the flaw size is comparable with the coil transducer [54]. Short and small diameter encircling coil probes provide higher sensitivity to small cracks than long and big diameter probes. Grimberg *et al.* took this relationship between coil size and sensitivity into account and proposed a method for reconstructing the flaw in order to determine the crack’s depth [54]. The disadvantage is that the coil sizes must adapt to the size of the tubes or bars being produced.

Coil probes provide high sensitivity to defects when eddy current flow is drastically changed. This means that encircling coils are optimized for detecting short discontinuities parallel to the axis of the inspected tubes or bars. Differential encircling probes only detect discontinuities when a long crack that is parallel to the major axis enters and leaves the probe.

To detect long discontinuities over their full length, pancake-type rotating probes are designed. They are able to detect as small as 50 μm. Pancake-type probes scan smaller areas than encircling coils which means the pancake-type probes are more sensitive [73].

Automatic scanning is widely used in production lines. Automatic inspection using pancake-type probes is complex, because they require rotating systems. The automatic scanning using encircling probes is simpler than using pancake-type probes because they are static. Encircling probes provide more control over production quality at very high speeds up to 150 m/s.

Segment coil probes are specifically designed for controlling the weld seam of welded pipes [59]. The sensitivity of segment probes is higher than encircling probes as they limit the scanning surface to the weld area, whereas encircling probes can scan 360 degrees.

Horseshoe-shaped coils are useful in the detection of laminar flaws that pancake-type coils cannot detect. Spiral coils provide high sensitivity and arrays of coils permit high-speed inspection and obtain high space resolution, reducing the coil size [62].

In general, the advantages of using coils as sensors for the eddy currents are the simplicity of their construction, the huge dynamic range and the possibility of focusing the sensor [48]. Some disadvantages are the high induction voltage at the start of the signal [48] and the fact that they are difficult to make smaller [17].

Other magnetometers can be used instead of pick-up coils. Hall sensors are magnetic-field sensors whose dynamic range is not large enough for some applications [48]. SQUIDs are difficult and expensive [48], although they provide very low field noise to the range of $\text{fT}/\sqrt{\text{Hz}}$ when compared to induction coils that have field noise of about $1\;\text{nT}/\sqrt{\text{Hz}}$ [55]. Many authors find the structure and characteristics of magnetoresistive sensor attractive for non-destructive evaluation because of their micro size, high frequency operation and high sensitivity [68].

## 5. Eddy Current Equipment

This section describes some types of eddy current testing equipment. Manufacturers of eddy current testing equipment offer a wide variety of equipment, from basic equipment to advanced equipment that is designed to satisfy the highest requirements. Basic eddy current equipment is used for sorting test pieces into two categories: good or bad pieces. They are low-cost and have only the essential controls and basic displays and may permit a connection to an oscilloscope [74]. Basic instruments have one or two physical channels that can be time multiplexed to increase their functionality. Instruments that satisfy basic requirements in production line can detect composition in alloys, measure parameters—such as hardness, case depth and temper—in heat treatments, measure sinter density and detect structure variations [74]. Different enclosures are typically available. RS232/V24 interfaces permit communication with main frame computers. Some opto-isolated inputs and outputs are available for the connection of other systems.

**Figure 29.**
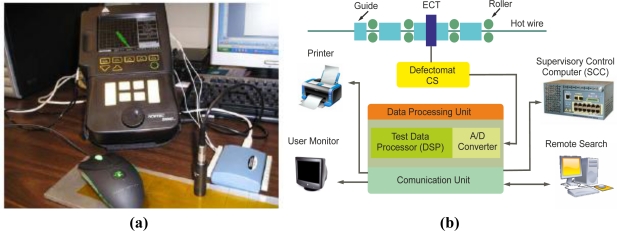
**(a)** Portable eddy current testing instrument [78]. **(b)** Block diagram of the overall system for hot wire testing (adapted from [6]).

Manufacturers also make portable instruments which contain the screen, controls and connectors in a compact enclosure as Figure 29(a) illustrates. Compact instruments may be operated via a standard LAN (Ethernet, TCP/IP) connection or together with other systems via one single screen [75].

High functioning eddy current instruments provide higher data processing capability and more physical channels than basic instruments. The top ten instruments permit hot wire testing at production speeds of up to 150 m/s, providing very high spatial resolution, as seen in the system represented in Figure 29(b). They also allow network integration in the production process and multi-frequency operation bands for calibration and testing [60]. Many top-ten instruments provide several USB 2.0 interfaces, Ethernet interfaces and printer connections to generate hard copies of test results. High-end eddy current instruments have more opto-isolated interfaces than basic instruments, up to 128 inputs and outputs for connecting a PLC to control automatic systems. Unlimited configurations can be stored on and loaded from hard disks [59].

Manufacturers construct multichannel eddy current instruments for rotating systems to detect longitudinal defects at speeds of up to 12,000 rpm. Many rotating systems are available with lift-off compensation that provides an extremely reliable method for defect detection [59].

Modern instruments generate frequencies in the range from kHz to MHz and permit the application of discrete signal processing, such as filtering and numerical demodulation. Many modern instruments include the impedance on XY plotters and also the X and the Y plot *vs.* time on LCD screens (or computer monitors if they are computer-enabled). Alarm settings on XY plotters permit users to activate programmable outputs that can activate light and sound alarms to alert the operator when cracks are present [75]. Instruments permit automatic scanning which activates automatic mechanisms to sort flawed pieces or activates paint markers. They also offer very high test speeds that can reduce the occurrence of human errors [76].

Several eddy current instruments are available with computer connections that vastly increase their capabilities to search, visualize and analyze eddy current inspection data [6,75]. Computers can receive data from multiple channels and real-time processes. Computers can also extract parameters of interest from signals, generate reports and store the signal from eddy current testing instruments in order to post-process the data. Some authors, such as Fahmy [76], Stander *et al.* [61], and Rao *et al.* [77], have published papers relating to computer-controlled eddy current systems.

## 6. Applications of Eddy Current Testing

Eddy current testing has a wide variety of applications. The most important applications and research are described in this section.

Eddy-current testing provides a high level of sensitivity for material identification and for the characterization of the microstructure state [22]. Absolute coil probes can measure physical parameters via the impedance which is related to the electrical conductivity and magnetic permeability of test pieces. Because of the relation between hardness and these variables, eddy current testing permits heat damage detection and heat treatment control. Mercier *et al.* published their research on hardness testing for the evaluation of steel decarburizing [8]. Eddy current techniques also take advantage of lift-off variation to measure the coating thickness of non-conductive materials or the oxide thickness of conductive materials [9].

Eddy current testing has many applications as a method of crack detection. The aeronautical and nuclear industries have invested many resources in the development eddy current testing. Authors such as Morozov *et al.* [10] and Thollon *et al.* [15] have worked with eddy current testing in the field of aeronautics. Others like Chen *et al*. and La *et al.* have used eddy current testing to research steam generator tubes in the nuclear industry [64,79].

In the metallurgical industry, authors such as Stander *et al.* have conducted research testing green-state powdered materials [61]. Manufacturers also offer special solutions for extra fine wires of tungsten and molybdenum testing up to 10 m/s [60]. In the field of transportation, researchers such as Pohl *et al.* have proposed railroad track surface testing at train speeds of 70 km/h [14].

Rotating inspection systems are used in wire drawing machines, copper tube winders or finishing lines in the bright steel sector [60] and are capable of finding longitudinal defects at very high speeds with a minimum depth of 0.05 mm [59,60].

In the field of hot eddy current testing, the inspection of different types of bars and profiles at temperatures of up to 1,200 °C can be performed using water-cooled probes [59,75]. This kind of inspection at high temperatures is useful for detecting these defects at an early stage before significant amounts of faulty material have been produced [75]. Testing of hot-wire line presents several difficulties such as low fill factor due to water cooling between the hot wire and the encircling coil and the necessity of high-speed data processing due to the very high speed of the line [6]. Eddy current testing is the only automated non-destructive test method capable of getting quality results at up to 150 m/s [7].

In production lines, defects can be either random or periodic in the material [75]. Random defects may indicate a poor overall quality of the material, suggesting deficiencies in the raw material or flaws in the general production process. Periodic defects that recur at regular intervals are likely to be generated by damaged rollers or guide rollers in the production line. Some researches devise techniques for detecting periodically occurring flaws based on the FFT technique [6]. Cracked rollers can be revealed by calculating simple equations using the speeds of the rollers and the sizes of their rolled wire [6].

The detection of residual stresses in engineering structures that can provide early indications of stress status and eventual failure is a rapidly growing area in non-destructive testing [80]. Eddy current coil probes can also detect very small stress variations in ferromagnetic steels due to the magneto-elastic effect based on the measurement of changes in impedance [20].

## 7. Conclusions

Nowadays, destructive or non-destructive techniques are more frequently used to test products due to the increase prevalence of quality controls. While destructive techniques verify only some samples that are destroyed and make some invalid in other industrial processes, we find non-destructive techniques more interesting than destructive ones since all production can be tested without permanent alterations.

This paper reviews the state-of-the-art methods of eddy current testing which is one of the most widely used non-destructive forms of testing. Eddy current testing permits crack detection and measurements that are beyond the scope of other techniques such as non-conductive coating thickness [9], alloy composition and hardness [8] in a large variety of materials. The only need is that the materials being tested must be electrical conductors where eddy currents can flow.

Eddy current sensors are insensitive to dirt, dust, humidity, oil or dielectric material in the measuring gap and have been proven reliable in a wide range of temperatures [28]. Coil probes are the most widely used type of sensors, and standard coils can be used in a wide range of applications [74].

Although eddy current testing has been developed for several decades, research into developing new probes, techniques and instrumentation is currently being conducted by manufacturers and research groups around the world in order to satisfy the increasingly higher quality standards required in almost every industry. These days, scientists are trying to develop new coil probes and research the use of other magnetometers such as superconducting quantum interference devices (SQUIDs), Hall-effect and magnetoresistive sensors that also provide very interesting responses.

The review of research into electromagnetic models and powerful simulators that help the probe designer to solve the forward [58] and inverse [25] flaw-probe problems is essential to optimal crack detection in terms of sensors and the operating variables such as frequency and signal-to-noise ratio.

Eddy current testing is a versatile technique that makes possible the hot eddy current testing of semi-finished products such as wires, bars and tubes at temperatures of up to 1,200 °C [59,75] and at production speeds of up to 150 m/s [7]. Early detection of these defects in production lines can save large sums of money in the metal industry.

In conclusion, as researchers and developers of solutions based on eddy current testing, we have found that eddy current techniques can provide the industry with reliable quality control systems. Although there are excellent improvements due to the effort of the many scientists during the last several years, we believe that more research in eddy current techniques, in terms of sensors, equipment and signal processing, will lead to even more applications of these techniques.
